# Acute renal failure after kidney transplantation due to mizoribine-induced ureteral stones

**DOI:** 10.1186/s12882-023-03418-5

**Published:** 2024-01-03

**Authors:** Mao Ding, Hongchao Zhao, Hengcheng Zhu

**Affiliations:** Department of Urology, Renmin Hospital of Wuhan University, No.238 Jiefang Road, Wuchang District, Wuhan, 430060 Hubei China

**Keywords:** Mizoribine, Kidney transplantation, Ureteral stones, Acute renal failure

## Abstract

**Introduction:**

Mizoribine (MZR) is used to prevent rejection reactions after kidney transplantation and increase the risk of hyperuricemia. There is a lack of reports of MZR-induced ureteral stones after kidney transplantation. The surgery treatment of ureteral stones in transplanted kidney is a challenging clinical issue that should only be performed by experienced urologists at professional centers. It is very important to have a thorough understanding of the patient's medical history, analyze the causes of stone formation, and choose a reasonable treatment plan based on the characteristics of the stones. The case report is aim to emphasize the recognition of the possibility of mizoribine-induced ureteral uric acid stones in transplanted kidney and to avoid unnecessary surgery.

**Case presentation:**

A patient after kidney transplantation was diagnosed with acute renal failure caused by ureteral stones. The medical history, CT images of the renal graft, the results of laboratory test and stone composition analysis were provided. Based on medical history and laboratory test results, it was determined that the ureteral stones of renal graft was induced by MZR. To our best knowledge, this is the first report of MZR-induced stones in transplanted kidney and ureters. It was completely cured by urinary alkalinization, avoiding surgery treatment. We summarize the characteristics, treatment and methods for preventing the formation of uric acid stones of patients with MZR.

**Conclusion:**

By analyze our case report, it shows that acute renal failure with ureteral stones after kidney transplantation can caused by MZR. Urinary alkalinization for MZR induced uric acid stones is simple and effective.

**Supplementary Information:**

The online version contains supplementary material available at 10.1186/s12882-023-03418-5.

## Introduction

Kidney transplantation was first successfully performed in the 1950s and is an important treatment method for most patients with end-stage renal failure. Ureteral obstruction can cause acute renal failure in the transplanted kidney [1]. Graft renal and ureteral stones are uncommon [2]. Mizoribine (MZR) prevents rejection during kidney transplantation by inhibiting lymphocyte proliferation and differentiation. To date, the characteristics and treatment of acute renal failure after kidney transplantation due to MZR-induced ureteral stones have not been reported. Extracorporeal shockwave lithotripsy (ESWL) and endoscopic surgery are commonly used to treat renal graft stones; however, patients undergoing these procedures face an increased risk of infection and severe adhesions [3]. This case report aimed to emphasize the possibility of MZR-induced ureteral stones in transplanted kidneys. Further, we summarize the characteristics and treatment of MZR-induced ureteral stones in transplanted kidneys, as this treatment may allow patients to avoid unnecessary surgery and the possibility of infection.

## Case presentation

A 39-year-old male patient presented to our department after kidney transplantation on November 20, 2021, with a two-day history of elevated serum creatinine levels, accompanied by intermittent abdominal pain and hematuria. Three years prior, the patient had received a kidney allograft from a deceased kidney donor, followed by standard triple immunosuppressive therapy consisting of mycophenolate mofetil (MMF), tacrolimus (Tac), and methylprednisolone (methylprednisolone) for chronic kidney disease (CKD) stage IIIb caused by chronic glomerulonephritis. One month after the transplant, the patient’s post-transplant serum creatinine level was 1.2 mg/dL (563.1 μmol/L). During follow-up, the patient's serum creatinine (SCr) level was 1.1–1.3 mg/dL (98.3–115.2 μmol/L), serum uric acid (UA) level was 4.9–6.9 mg/dL (291.4–413.8 μmol/L), and urine protein was < 0.2 g/24 h. Two weeks prior, the patient had begun receiving mizoribine as an alternative to MMF for immunosuppressive therapy owing to diarrhea.

Urinalysis revealed red cells, white cells, and pH less than 5.0. Urine cultures were sterile. The SCr level was 6.3 mg/dL (563.1 μmol/L) and the UA level was 31.9 mg/dL (1901.0 μmol/L). The blood potassium level was 4.15 mmol/L, and the blood calcium level was 2.35 mmol/L.

**Fig. 1:**
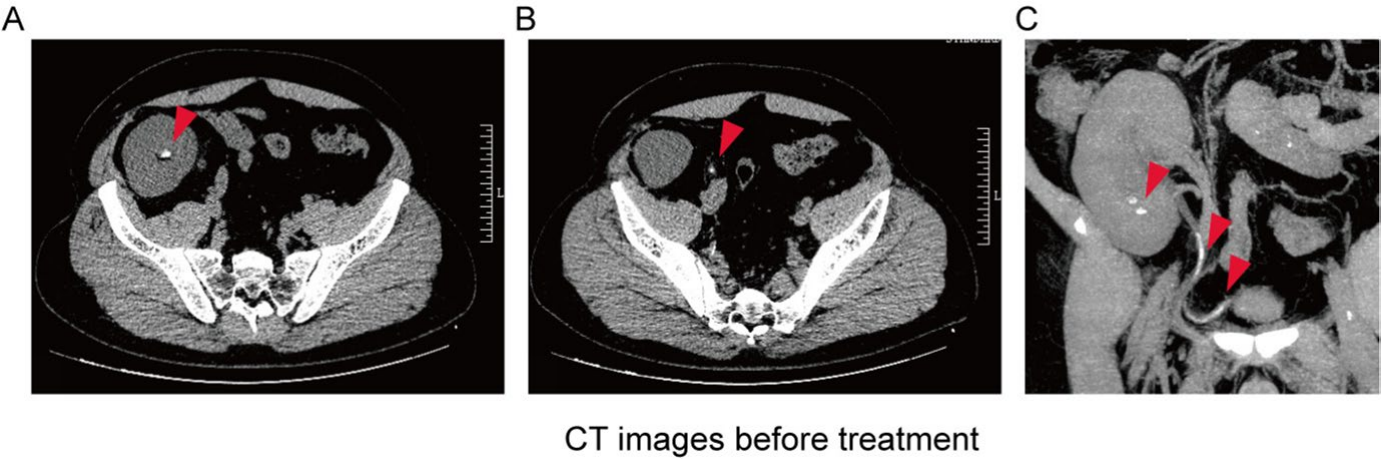
**A** CT image showed that the patients had a transplanted kidney stone before treatment. **B** and **C**. CT image showed that the patients had ureteral calculus before treatment

The 24-h urinary excretion of uric acid was 105.4 mg/dL (6269.4 μmol/L). Ultrasound (US) examination showed no sign of rejection, but allowed detection of moderate hydronephrosis in the transplanted kidney. CT revealed hydronephrosis and kidney stones (6.9 mm and 5.7 mm) and ureter allografts (33.5 mm and 37.4 mm) with 135–336 HU (Fig. 1). Based on clinical data, acute renal failure with obstruction caused by mizoribine-induced uric acid stones was suspected.

**Fig. 2:**
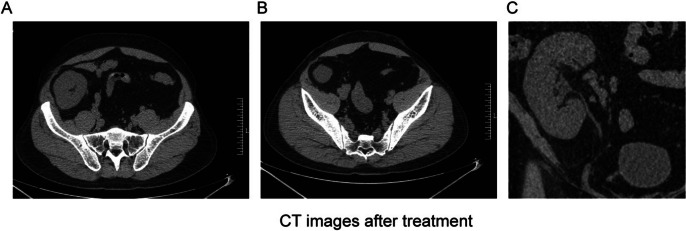
**A**. CT image showed that the transplanted kidney stone was completely cleared after 7 days treatment. **B** and **C**. CT image showed that ureteral calculus were completely cleared after 7 days treatment

**Fig. 3:**
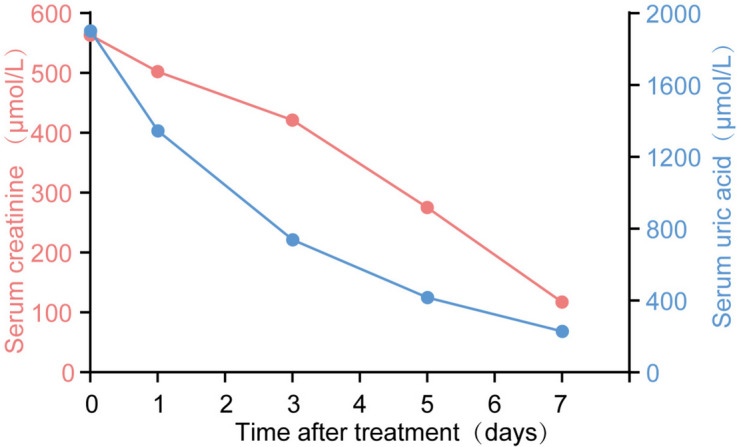
The variation trend of SCr and UA after urinary alkalinization for 7 days

Owing to the large axial diameter of the ureteral calculus, transurethral ureteroscopic lithotripsy and ESWL were not performed. Mizoribine was discontinued and supportive measures were initiated, including high fluid intake and oral potassium citrate (60 mEq). After three days of treatment, small crystals were discharged from the urethra. Constituent analysis using Fourier Transform Infrared Spectroscopy (FTIS) revealed that the stones were composed of uric acid. After seven days of treatment, CT revealed complete dissolution of the stones in the kidney and ureteral allograft (Fig. 2). The patient’s SCr returned to 1.2 mg/dL (107.4 μmol/L) and UA returned to 5.3 mg/dL (315.6 μmol/L). (Fig. 3) The blood potassium level is 4.60 mmol/L, and the blood calcium level is 2.29 mmol/L. During a two-year follow-up period, the patient continued to receive immunosuppressive therapy with mizoribine, tacrolimus, and methylprednisolone. He was administered potassium citrate to maintain urine pH between 6.5 and 7.0. The patient's SCr was 1.1–1.4 mg/dL (101.1–121.9 μmol/L), the UA was 4.4–6.8 mg/dL (262.5–404.7 μmol/L), and urine protein was < 0.2 g/24 h. The patient did not experience acute renal failure or ureteral stones.

## Discussion

Mizoribine is a purine synthesis inhibitor that exerts immunosuppressive effects by inhibiting lymphocyte proliferation and differentiation, and is used to prevent rejection reactions during kidney transplantation. Owing to the low incidence of adverse events such as bone marrow suppression, gastrointestinal discomfort, and viral infections, this drug is typically used as an alternative to mycophenolic acid (MPA) drugs. Previous studies have reported that mizoribine increases serum uric acid levels, with an incidence rate of approximately 32.0–87.5% [4, 5]. Further, it competitively inhibits inosine monophosphate dehydrogenase (IMPDH), leading to an increase in purine alkaloids and subsequent production of more uric acid; this ultimately results in elevated serum uric acid levels [6, 7]. Literature has reported that hyperuricemia can lead to deterioration of the renal glomerular filtration rate in kidney transplant patients [8]. However, to the best of our knowledge, acute renal failure in transplanted kidneys caused by mizoribine-induced uric acid stones has not been reported. When the pH is less than 6.0 and the solubility of uric acid in urine decreases significantly, uric acid crystallization occurs and uric acid stones are formed, resulting in obstruction of the transplanted kidney [9]. In this study, the 24-h urinary uric acid excretion of the patient increased significantly (6269.4 μmol/L) and the pH of urine decreased to less than 5.0 after mizoribine administration. These factors contributed to acute obstruction of the transplanted kidney in this study. However, we should emphasize that the presence of high uric acid levels does not always lead to the formation of kidney stones. Conversely, not all kidney stone patients necessarily have high uric acid levels.

In this case, the patient who presented with diarrhea was subsequently switched to mizoribine instead of MMF two weeks before admission to the hospital. At the time of initial evaluation, routine renal ultrasound examination did not reveal any evidence of kidney stones, ureteral dilation, or renal pelvis dilatation two weeks prior. Furthermore, it is generally believed that uric acid stones, which are relatively loose and recently formed, dissolve more easily in alkalized urine. After seven days of urine alkalization therapy, CT scans indicated that uric acid stones in the renal pelvis and ureter had rapidly disappeared, suggesting that these stones may have formed recently.

Currently, the main reported methods for treating transplant kidney stones include extracorporeal shock wave lithotripsy (ESWL), retrograde ureteroscopy, percutaneous nephrolithotomy (PCNL), and minipercutaneous nephrolithotomy (MPCNL) [10]. Owing to the immunodeficient status of the patient and the anatomical position of the transplanted kidney (in the iliac fossa), the management of transplanted kidney stones is particularly challenging. The ureter of the transplanted kidney is often anastomosed at the dome of the bladder, significantly increasing the difficulty in identifying and inserting the ureteroscope during retrograde ureteroscopy. Owing to the increased risk of infection, severe adhesions, wound infections, and delayed wound healing, only experienced urologists at professional centers should be allowed to perform surgery [11]. In this case, the axis diameter of the patient's ureteral stone was large and the line of the transplanted ureter was tortuous, limiting the use of commonly used surgical treatments to relieve the obstruction. Based on the medical history and laboratory test results, we concluded that the stone was a mizoribine-induced uric acid stone. These stones are primarily caused by excessively acidic urine and can be dissolved by urinary alkalinization, with medications such as potassium citrate [12]. We believe that urinary alkalinization should be considered as treatment for mizoribine-induced uric acid stones in transplanted kidneys, as this technique can prevent surgical damage to the transplanted kidney function and reduce the risk of infection.

Nephrostomy is a crucial treatment for transplanted kidney patients with acute kidney failure due to urinary obstruction. This procedure can effectively relieve urinary obstruction, reduce pressure within the kidneys, and prevent severe renal failure. In patients with acute renal failure caused by mizoribine-induced uric acid stones who consent to undergo nephrostomy therapy, we recommend its initial use to relieve obstruction, followed by subsequent urine alkalinization or surgical treatment. However, in this case, the patient declined nephrostomy and achieved favorable treatment outcomes with urinary alkalinization.

After high-dose mizoribine treatment in kidney transplant patients, uric acid-lowering drugs, such as febuxostat, benzbromarone, or allopurinol, are often used. Considering that excessive uric acid production after mizoribine administration leads to elevated serum uric acid levels, we recommend drugs that reduce uric acid production, such as febuxostat. However, cardiovascular safety has been a concern in many studies [13, 14], and the risk of cardiovascular death is dose-dependent [14]. Therefore, caution should be exercised when using febuxostat in kidney transplant patients with a history of atherosclerosis, myocardial infarction, or congestive heart failure. Benzbromarone can also be used to control uric acid levels. However, if this is done, we strongly recommend alkalizing the urine and monitoring the urine pH during administration; uric acid has higher solubility in urine with higher pH, and is less likely to form crystals. In the present case, the patient continued to receive benzbromarone for hyperuricemia due to atherosclerosis and coronary heart disease. After urinary alkalinization with potassium citrate, uric acid stones did not recur during a follow-up period of approximately two years, and the patient’s serum creatinine was maintained between 95 and 115 μmol/L.

Notably, the blood potassium levels in our patient remained within the normal range and the patient demonstrated the ability to urinate independently; this suggested an incomplete obstruction of the transplanted kidney. However, in cases of acute transplantation renal dysfunction, administration of potassium citrate poses a potential risk of hyperkalemia. This further increases the risk of cardiac arrhythmias and exacerbates renal function decline. Hence, we recommend administration of sodium bicarbonate to alkalize urine as a safer and more appropriate treatment option during acute renal failure in transplant recipients. When renal function has recovered and the obstruction is relieved, potassium citrate can be considered to raise the pH of the urine under regular monitoring of serum potassium levels and renal function.

## Conclusion

In summary, this case report shows that mizoribine-induced UA stones can cause acute renal failure with obstruction after kidney transplantation. Urinary alkalinization for mizoribine-induced uric acid stones is simple and effective, and can prevent surgical damage to the transplanted kidney and reduce the risk of infection. pH screening should be performed in all kidney transplant patients undergoing mizoribine treatment.

### Supplementary Information

**Additional file 1: Table1.**The characteristics of Corynebacterium-related encrusted uretero-pyelitis and Mizoribine-induced encrusted ureter. 

## Data Availability

The data that support the findings of this study are available from corresponding author and Renmin Hospital of Wuhan University.

## Abbreviations

MZR: Mizoribine
ESWL: Extracorporeal shock wave lithotripsy
MMF: Mycophenolate mofetil
Scr: Serum creatinine
UA: Serum uric acid
US, e: Ultrasound
MPA: Mycophenolic acid
IMPDH: Inosine monophosphate dehydrogenase
PCNL: Percutaneous nephrolithotomy
MPCNL: Mini-percutaneous nephrolithotomy
