# Supplementary material for: Acute renal failure after kidney transplantation due to mizoribine-induced ureteral stones
Source: BMC Nephrol. 2024 Jan 3;25:5. doi: 10.1186/s12882-023-03418-5 (PMC10765741; doi:10.1186/s12882-023-03418-5)
Supplement: Supplementary file 1 — Additional file 1: Table1.The characteristics of Corynebacterium-related encrusted uretero-pyelitis and Mizoribine-induced encrusted ureter. [file 12882_2023_3418_MOESM1_ESM.docx]

Table1. The characteristics of Corynebacterium-related encrusted uretero-pyelitis and Mizoribine-induced encrusted ureter

| Item | Corynebacterium-related encrusted uretero-pyelitis | Mizoribine-induced encrusted ureter |
| --- | --- | --- |
| Predisposing factors | immunocompromise, urogenital tract trauma | taking Mizoribine |
| Necessary conditions | Corynebacterium infections | hyperuricemia |
| Characteristics of CT | hydronephrosis and calcifications within transplanted ureter and kidney | hydronephrosis and calcifications within transplanted ureter and kidney |
| pH of Urine | alkaline urine (PH 7-9) | acidic urine (PH＜5) |
| Composition of the stones | carbonate apatite, ammonium urate,  ammonium–magnesium phosphate hexahydrate | uric acid |
| Conservative treatment | administration of antibiotics, urinary acidification | Stopped administration of mizoribine, urinary alkalinization and high fluid intake |
